# Behavior of Grouted Sleeve Splice for Steel Profile under Tensile Loadings

**DOI:** 10.3390/ma13092037

**Published:** 2020-04-27

**Authors:** Feng Lin, Peng Zhao

**Affiliations:** 1 Key Laboratory of Performance Evolution and Control for Engineering Structures (Tongji University), Ministry of Education, Shanghai 200092, China; 2 Department of Structural Engineering, Tongji University, Shanghai 200092, China; zhaopengld@163.com

**Keywords:** steel profile, grouted splice, grout-filled sleeve, anchorage length, FEM model

## Abstract

Two groups of grouted sleeve splices for steel profile were tested to investigate their tensile behavior, serving as pilot studies for novel prefabricated reinforced concrete shear wall structures. In the first group, four splice specimens with different embedded lengths of steel profile were monotonic tensile loaded to determine an appropriate anchorage length. In the second group, three splice specimens with a conservative anchorage length of steel profile were tested under repeated tensile loading, cyclic loading at high stress, and cyclic loading at large strain, respectively. Parametric studies were then conducted on sleeve thickness, grout strength, offset of steel profile, and misalignment of steel profile using finite element method (FEM)-based models. The results show that the splices in the second group behaved well with minor residential deformation and almost no pinching effect. The optimized sleeve thickness could be appropriately determined using FEM models. The compressive strengths of the grout exceeding a threshold value and the offset of steel profile had minor influence on splice behavior, while the misalignment of steel profile had a pronounced effect.

## 1. Introduction

Precast concrete structures are constructed worldwide because they speed up construction, require less labor, and minimize the impact on the environment compared to cast-in-situ concrete structures [1,2]. Consequently, precast construction is extensively used in many countries in commercial and institutional buildings [3] as well as in bridge engineering [4]. A majority of precast components are fabricated in factories, transported to the construction site, and connected to implement structural integrity. To achieve this process, using grout to connect reinforcing steel bars in adjacent components is regarded as a preferred technology due to controllable tolerance and no need to weld at construction site.

**Figure 1:**
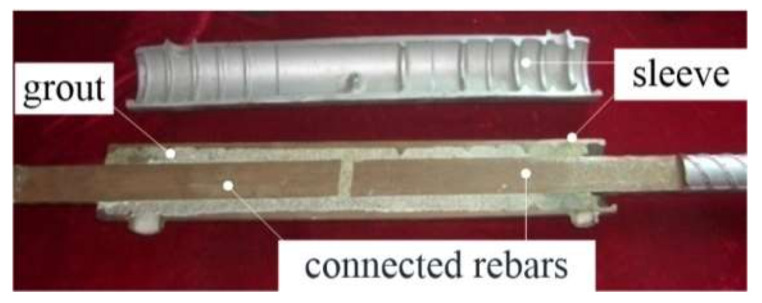
A cross section of a grouted sleeve splice of typical configuration to connect reinforcing steel bars.

Two main technologies can be identified for connecting rebars in precast concrete structures, i.e., grouted sleeve splice and grouted duct connection. For the grouted sleeve splice, Figure 1 illustrates a cross section of such splice of typical configuration. Two reinforcing steel bars were collinear and inserted into the sleeve. After that, commercially available grout, which is specifically prepared for the grouted sleeve splices, was poured into the sleeve using dedicated facilities. The grout is cement-based and featured with high fluidity, slightly expanding, and high early strength. Over the last few decades, many experimental and numerical studies have been conducted to understand the failure modes, influencing factors and structural performance of the grout-filled splices [5,6,7,8,9,10,11,12,13]. These studies have revealed that rebar fracture and rebar pull-out for splices under tensile loading were the most common failure modes that occurred in tests. The critical factors that influenced the structural performance of the splices were proved to be the embedded length of the rebar, sleeve configuration and grout strength. Test results also have indicated that the ultimate strengths and deformability of the splices is slightly lower than those of the connected rebars, respectively. The grout-filled sleeve provides radial confinement to enhance the bond through interaction among the sleeve, grout and rebars, resulting in a transfer of tensile or compressive force between the two discontinued rebars. For the grouted duct connection, non-contact lap splices are used for rebars [14,15,16]. The rebars protruding from one precast component are grouted into corrugated steel ducts encased in the other one. When using these connections in precast columns, two smaller-diameter rebars, adjacent to each duct, are usually present [16].

**Figure 2:**
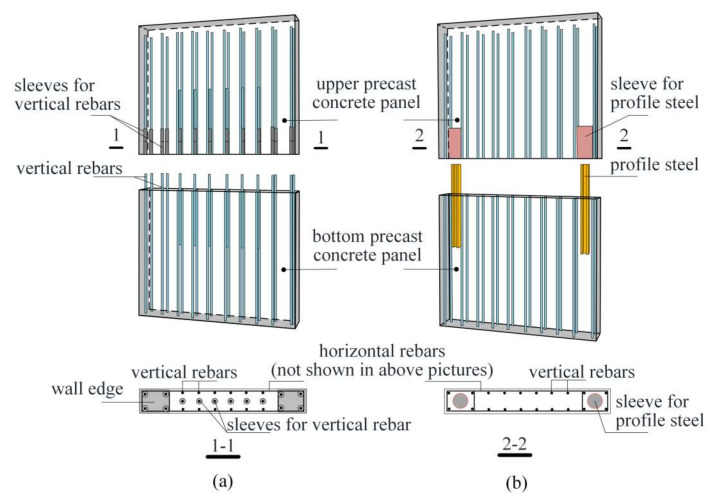
Precast concrete shear walls with (**a**) splices to connect vertical rebars; and (**b**) splices for steel profiles.

Grouted sleeve splices are often used in precast concrete shear walls due to efficient construction and economic reasons. A typical example of using grouted sleeve splices to connect an upper and bottom precast concrete panel at a construction site is illustrated in Figure 2a. Vertical rebars protrude from the bottom fixed panel. The upper movable panel contains the embedded sleeves at the same location as the rebars, and then adjusts to an appropriate location so that the rebars in the bottom panel can be accurately inserted into the sleeves. Afterwards, the upper panel is temporarily fixed, and grout is poured into the sleeves. Eventually, the upper and bottom panels will vertically connect to form a horizontal joint after the grout hardens.

However, a trouble occasionally occurs in construction site when inserting a row of rebars into the sleeves due to limited construction tolerance [7]. For reasonable transfer of force, the sleeve diameter is designed slightly larger (e.g., a few millimeters) than the matched rebar diameter. Currently in China, precast concrete shear walls have their lengths commonly ranging from 3 to 4 m, with the rebars arranged in a single row and a rebar number of at least six (excluding the rebars embedded in the wall edges as illustrated in Figure 2a) after optimization. In fact, assembly accuracy can be affected by many factors, including manufacturing error and possible rebar collision between different components during the transportation process. Consequently, many efforts are made to correct the defective rebar at the construction site, resulting in a potential safety risk and reduction of assembly efficiency [17,18].

A novel alternative to mitigate this problem is proposed in this study [19]. The basic idea is to change the vertical rebar connection mode from the conventional “distribution along the wall” to “concentration in the wall edges.” As illustrated in Figure 2b, sleeves and steel profiles are embedded in the upper and bottom panels, respectively, and are connected by pouring grout. In Figure 2b, the grout also fills the horizontal joint between the two panels as is the case in conventional precast concrete shear walls using grouted sleeve splices for rebars. The cross sectional area of the steel profile can be determined based on the principle of “equivalent bearing capacity.” In other words, the bearing capacity of a conventional precast concrete shear wall and the proposed one should be approximately equivalent in terms of bending and direct shear resistances. Conceptually, the proposed precast concrete shear wall has the advantages of reasonable structural form to resist bending moment and direct shear, high defect tolerance, and efficient assembly.

The structural performance of the novel precast concrete shear wall should be comprehensively investigated. As a pilot research, this study aimed to investigate the behavior of the grouted sleeve splice for the steel profile (to be referred to as splice from this point forward) under different tensile loadings by means of experimental and numerical studies. The tensile loadings contained four loading schemes, i.e., monotonic tensile loadings (MT), repeated tensile loading (RT), cyclic loading at high stress (HS), and cyclic loading at large strain (LS). The last three loading schemes involve a splice under service loads, winds and frequent earthquakes, and rare earthquakes, respectively. After that, finite element method (FEM)-based models were built, validated, and used to perform parametric studies to provide further understanding as to how the critical parameters affect the tensile behavior of the splices.

## 2. Experimental Program

### 2.1. Specimens

The prototype of a concrete shear wall is cast-in-place with a thickness of 200 mm and a length of 3200 mm including two 400-mm-long wall edges. The wall was seismically designed and typically reinforced in accordance with Chinese code GB50011 [20]. That is, the wall body, excluding wall edges, has a vertical reinforcement ratio of 0.25% for distributed reinforcing steel bars, and each wall edge was vertically reinforced with six 12-mm-diameter rebars. The bending bearing capacity of the prototype wall is 4752 kN·m with a compressive strength of concrete of 26.8 MPa, nominal strength of the reinforcing steel bars of 360 MPa and an axial compression ratio of 0.15. For the novel precast concrete shear wall with identical geometry to the prototype wall, the cross sectional area of each steel profile was calculated to be about 1280 mm^2^, based on the “equivalent bearing capacity” principle of bending moment, which takes into consideration the nominal strength of the steel profile of 345 MPa. An H-type cross section was used for the steel profile.

Table 1 presents the details of the splice specimens divided into two test groups, which were fabricated at different times. In the first test group of four splices, steel profiles were embedded in the grout with different embedded lengths *L* (i.e., *L* = 500, 400, 300, and 200 mm) to investigate the anchorage length of the steel profile. The specimens were statically tested under monotonic tensile loading. After completing the tests in the first group, three splices in the second test group with a conservative anchorage length of steel profile (i.e., 350 mm) were loaded under different loading schemes, as presented in Section 2.3.

**Figure 3:**
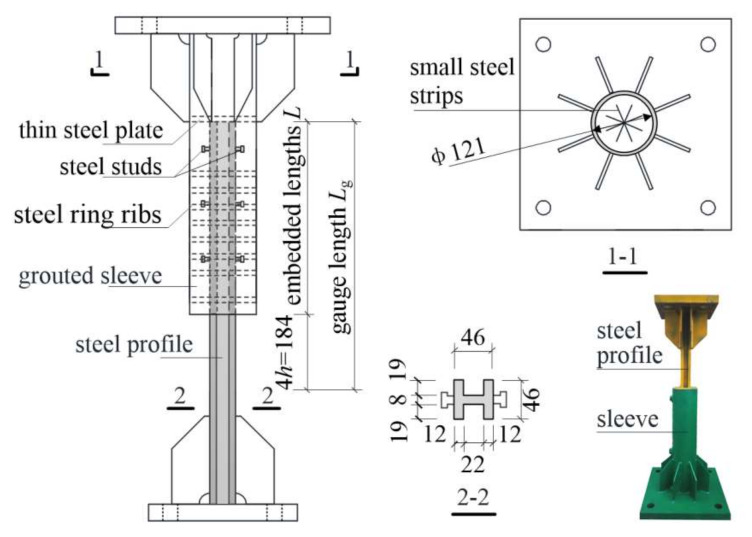
Geometry and configuration of specimens.

Figure 3 illustrates the geometry and configuration of the specimens. Sleeves were designed with the intent to provide (1) easy manufacturing, (2) avoidance of sleeve fracture under loading due to a weak cross section of the sleeve, and (3) avoidance of grout pull-out from the sleeve under loading due to grout-sleeve bond failure. To achieve this, common seamless steel pipes were used to produce the sleeves. The adopted steel pipes had an outer diameter of 121 mm and a wall thickness of 6 mm. Steel studs of 8 mm diameter and 15 mm length were welded on the surface of the steel profile, and 10-mm-diameter steel ring ribs were welded into the inner surface of the sleeves to enhance the grout-sleeve bond performance. In addition, for each specimen, a thin steel plate was welded near one of the sleeve ends to form a closed end. Small steel strips were welded on the inner surface of the plate to appropriately position the steel profile. When assembling, the steel profile was first inserted into the sleeve and appropriately positioned before the grout was directly poured into the sleeve. All specimens were cured outdoors until testing.

### 2.2. Materials

**Figure 4:**
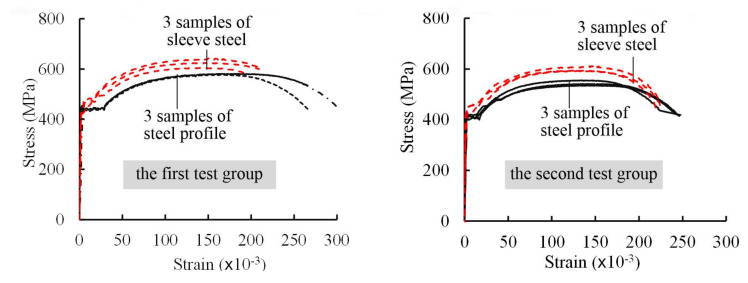
Stress-strain curves of steel profile and sleeve steel in the first and second test groups.

Table 2 and Figure 4 present the material properties and stress-strain curves for the steel profile and sleeve steel, respectively [21]. Table 3 presents the compressive strength and flexural strength of the grout which were tested on prism specimens (40 × 40 × 160 mm^3^) in accordance with JG/T408-2013 [22]. All data in the tables represent the mean values resulting from three material specimens. The high-strength grout used in the study was provided by Shanghai Livable Building Science & Technology Co., Ltd. The ready-to-mix grout was mixed using a water-to-grout ratio of 0.13 (32.5 L of water: 25 kg of grout) following the operation instructions provided by the manufacturer. The grout was fully mixed to form a well homogenous mixture before pouring.

### 2.3. Test Setup and Instrumentation

**Figure 5:**
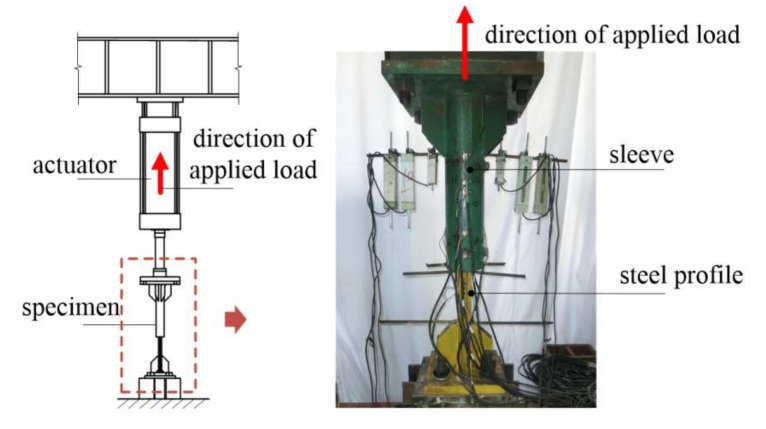
Test setup.

Figure 5 illustrates test setup using a 2000 kN servo-hydraulic actuator. The loading was statically applied and the forces were automatically recorded by a data acquisition installed in the actuator. Table 4 presents the loading procedures for loading schemes of monotonic tensile loading (MT) for the specimens in the first test group, and repeated tensile loading (RT), cyclic loading at high stress (HS), and cyclic loading at large strain (LS) for the specimens in the second test group. These loading schemes were in conformity with the Chinese code JGJ355-2015 [23] and similar to the international acceptance criteria AC133 [24]. As mentioned in Section 1, the last three loading schemes, i.e., RT, HS, and LS, relate to a splice under service loads, winds and frequent earthquakes, and rare earthquakes, respectively, as discussed in [10].

**Figure 6:**
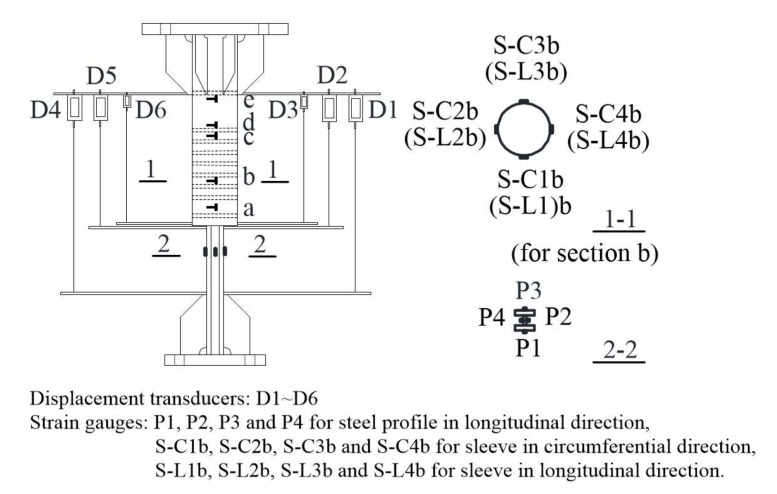
Arrangement of displacement transducers and strain gauges for Specimen RT350.

Figure 6 illustrates the arrangement of displacement transducers and strain gauges for Specimen RT350 as an example. The splice deformations within gauge length *L*_g_ (where *L*_g_ = *L* + 4 *h*, *L* and *h* = 46 mm denote an embedded length of the steel profile and cross sectional height of the steel profile, respectively) could be obtained using the mean value of the deformations measured using displacement transducers D1 and D4. As a result, the relative elongation at ultimate loads, *δ*_sgt_, is calculated by dividing the elongations at ultimate loads *u*_sgt_ by *L*_g_ using Equation (1):

(1) $$\delta_{\mathrm{sgt}}=\frac{u_{\mathrm{sgt}}}{L_{g}}$$

The sleeve deformation could be gained using the mean value of the deformations measured using displacement transducers D3 and D6. The relative deformations between the steel profile and grout due to the slip at the opening end of each sleeve could be calculated as the difference between sleeve deformation and the mean value of the deformations measured using displacement transducers D2 and D5. In addition, strain gauges were glued on the surfaces of the steel profile and sleeves to monitor their strain development and distribution.

## 3. Test Results

### 3.1. The First Test Group

**Figure 7:**
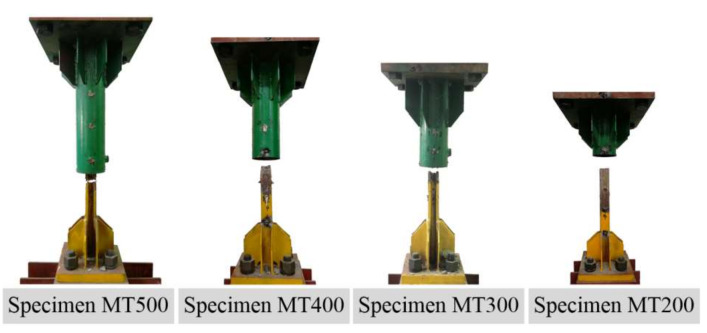
Failure mode of steel profile fracture for Specimens MT500, MT400 and MT300, as well as steel profile pull-out for Specimen MT200.

Two failure modes were observed and illustrated in Figure 7, i.e., steel profile fracture for Specimens MT500, MT400 and MT300, as well as steel profile pull-out for Specimen MT200. 

**Figure 8:**
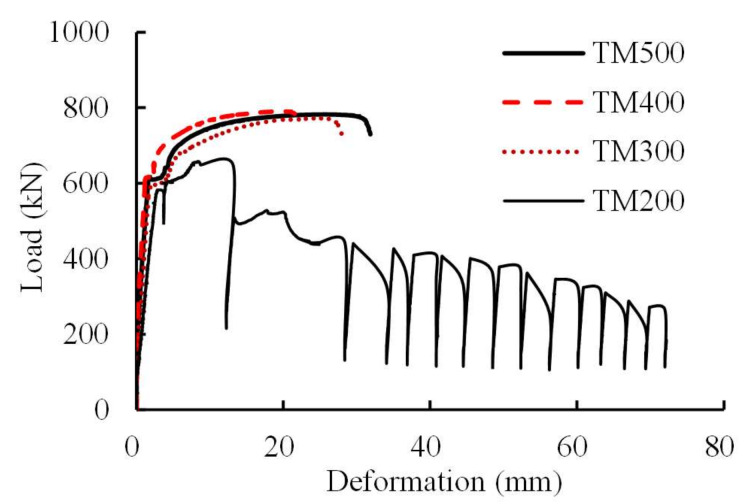
Load-deformation curves for Specimens MT500, MT400, MT300, and MT 200.

Figure 8 presents the load-deformation curves for Specimens MT500, MT400, and MT300, which can be identified by separating into three parts, i.e., pre-yield, yield, and post-yield parts. Before yielding, the specimens deformed slightly. Then, circular cracks occurred in the grout located in the opening end of the sleeves when the specimens approached yielding. After yielding, the grout at this location gradually dropped off and, finally, steel profiles fractured at ultimate loads. However, for Specimen MT200, the load–deformation curve oscillates after ultimate load as a result of repeated failure and compaction of local grout clung to studs. That is, the local grout could partially recover the resistance instantly after failure and compaction.

Table 5 presents the primary results of the specimens in the first test group. It was found that the average yield strengths and average ultimate strengths of Specimens MT500, MT400, and MT300 increased about 6% and 2% compared to those of the steel profile, respectively. This can possibly be attributed to the presence of weld seams, which enhanced the tensile resistance of the steel profile. However, the relative elongations at ultimate loads were significantly less than those of the steel profile, i.e., approximately 5.2% vs. 16.6%. This is a common observation for the grouted splices of reinforcing steel bars [6,10,23] and is mainly due to the weak deformation capacity of the sleeve region, which is further discussed in Section 3.2.

Based on these results, the anchorage length related to the failure mode of steel profile fracture was between 200 and 300 mm for the used splices in the first test group. However, an anchorage length of the steel profile was conservatively taken as 350 mm in the second test group, considering the possible variation of the geometry, materials, and construction quality.

### 3.2. The Second Test Group

**Figure 9:**
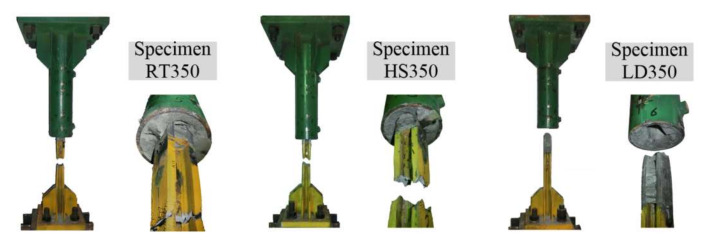
Failure mode of steel profile fracture for Specimens RT350, HS350 and LD350.

The failure mode of steel profile fracture was observed and is illustrated in Figure 9 for Specimens RT350, HS350 and LD350. 

**Figure 10:**
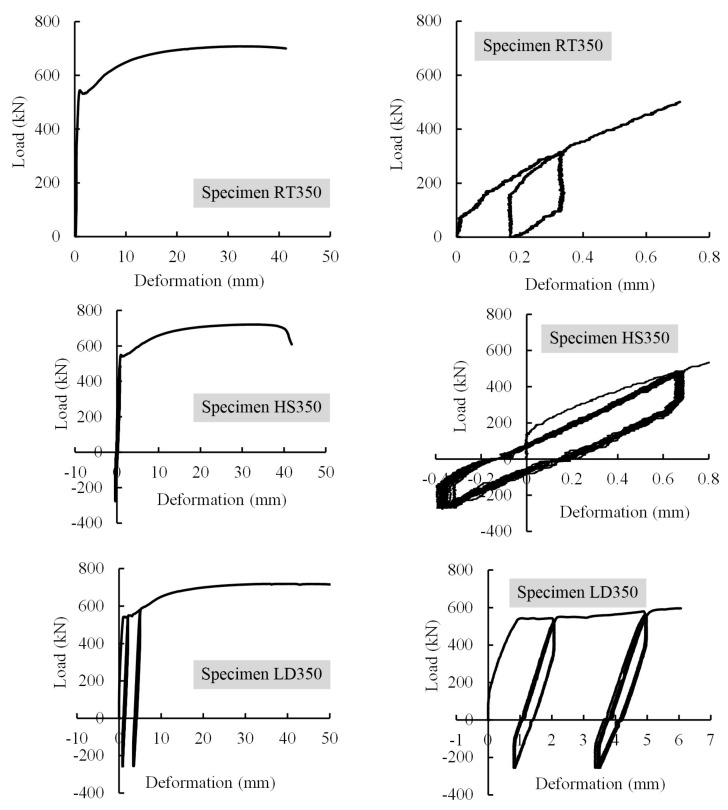
Load-splice deformation curves for Specimens RT350, HS350 and LD350.

Figure 10 and Table 6 present the load-deformation curves and primary results of these specimens, respectively. The observations during testing were similar to those of Specimens MT500, MT400, and MT300. Therefore, they are not presented for brevity. In general, results found that (1) the load-deformation curves can be identified as three parts, i.e., pre-yield, yield and post-yield parts; (2) the curve skeletons, ultimate strengths and relative elongations at ultimate loads of the three specimens were similar to each other. Similar results were also found in tests of grouted splices for reinforcing steel bars [10]; (3) residual deformations were minor, i.e., *u*_sgt_ = 0.165 mm for Specimen RT350, *u*_20_ = 0.185 mm for Specimen HS350, *u*_4_ = 0.285 mm and *u*_8_ = 0.410 mm for Specimen LD350; and (4) pinching effect was hard to identify for Specimens HS350 and LD350, which meant the slip between the steel profile and grout was insignificant.

**Figure 11:**
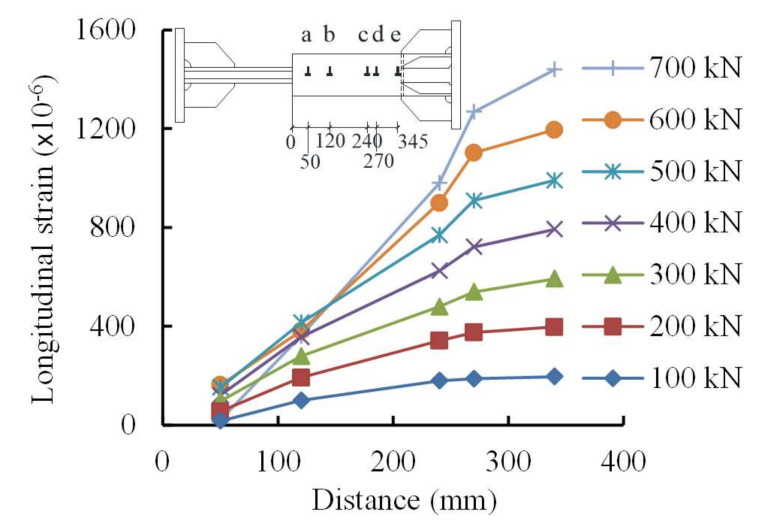
Longitudinal strains of sleeve for Specimen RT350.

**Figure 12:**
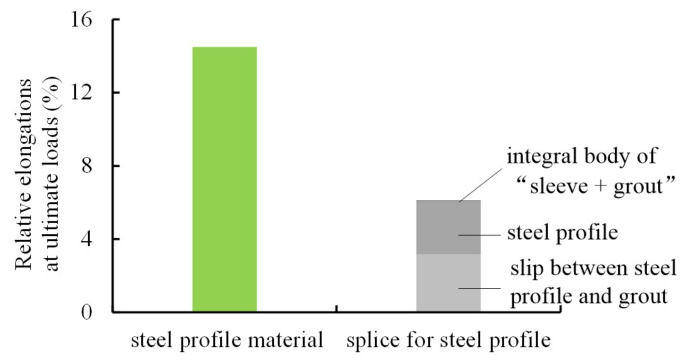
Comparison of deformation contribution within gauge length of Specimen RT350 at ultimate load.

In addition, responses of the specimens were investigated in terms of load-slip curves, strain development of the steel profiles, strain development of the sleeves, and deformation contributions for the specimens at ultimate loads. First, the slips between the steel profiles and sleeves for Specimens RT350, HS350, and LD350 at ultimate loads were 16.8, 16.4, and 22.6 mm, respectively. These slips were observed to contribute mainly by the slips between the steel profiles and grout, while the slips between grout and the sleeves were minor. The slip of Specimen LD350 was about 35% larger than those of Specimens RT350 and HS350 because the loading scheme of large deformation leads to slip increase. Second, the strain developments of the steel profiles revealed that the steel profiles were uniformly tensioned to failure and an eccentric loading was not observed. Third, sleeve strains of the three specimens were similar to each other and dominantly developed in the longitudinal direction illustrated in Figure 11 for Specimen RT350 as an example. Figure 11 shows that the sleeve behaved elastically and the maximum strain appeared in the closed end of the sleeve. In fact, sleeve deformation was a combination of three responses, i.e., longitudinal elongation, circumferential contraction due to the Poisson effect, and radial expansion after grout cracking [9,11]. Finally, Figure 12 compares the deformation contribution of Specimen RT350 at ultimate load. The relative elongation within gauge length is contributed by three parts, i.e., (1) the steel profile, (2) the slip between the steel profile and grout, and (3) the integral body of “sleeve + grout” segment including the slip between them. It was found that the deformation capacity of splices was contributed mainly by parts (1) and (2), and the contribution provided by part (3) was minor.

## 4. Numerical Studies

The purpose of this section was to further understand the behavior of the grouted sleeve splices for steel profile under tensile loadings and to optimize the geometrical configuration of the splices. Finite element method (FEM)-based models were built using Ansys/Ls-Dyna software [25] to conduct parametric studies.

### 4.1. FEM Models 

**Figure 13:**
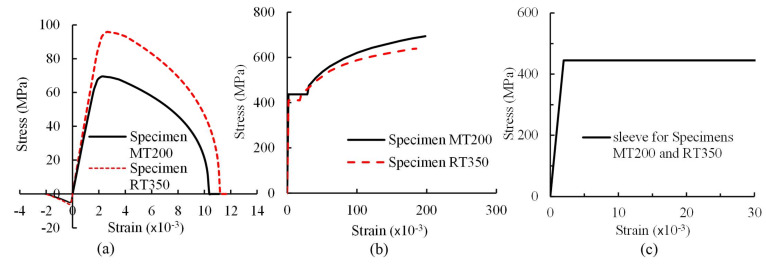
Material models for (**a**) grout; (**b**) steel profiles; and (**c**) sleeves.

FEM models were built with consideration of element types, material models, contact definition, and boundary condition. First, the eight-node solid element, Solid65, was used for the grout, steel profiles, and sleeves. One center integral point in solid elements with high computational efficiency was adopted, however, associated with undesired zero energy modes (hourglass modes). To eliminate these modes, a stiffness based hourglass control method (IHQ = 4) was used. Second, the Karagozian & Case (K&C) concrete model was adopted for modeling the concrete without consideration of strain rate effect. This material model was used by researchers to simulate the behavior of concrete under quasi-static loading with consideration of the confining effect under hydrostatic pressures [26,27]. Figure 13a illustrates the stress-strain relationship of the grout under uniaxial loading in the first and second test groups. Steel profile was modeled using the Cowper symbols model (*MAT_PIECEWISE_LINEAR_PLASTICITY) without consideration of strain rate effect. Steel profile failed when its axial ultimate strength was reached. Figure 13b illustrates the stress-strain relationship of steel profiles under axial tension for the specimens in the first and second test groups. For the steel sleeves, an elastic, perfectly plastic model with uniform material parameters was adopted because (1) the material properties of the sleeves used in the first and second test groups were almost identical to each other; and (2) steel sleeves did not reach yielding in tests. Figure 13c illustrates the stress–strain relationship of steel under axial tension for sleeves in elastic and plastic stages. Third, the contact between the steel profile and grout was simulated using the penalty function method (*CONTACT_AUTOMATIC_SURFACE_TO_SUR-FACE)with default parameters in software Ls-dyna to implement appropriate bond behavior. Finally, boundary condition was achieved by (1) restraining all degrees of freedom of the nodes in the closed end of the sleeves; (2) for the nodes in the longitudinal axis of the steel profiles, restraining their translational degrees of freedom in two directions other than in the loading direction; (3) for the nodes in the loading end of the steel profiles, restraining their translational and rotational degrees of freedom in two directions other than in the loading direction; and (4) applying displacement on the nodes of the steel profiles in the loading end along its longitudinal axis.

**Figure 14:**
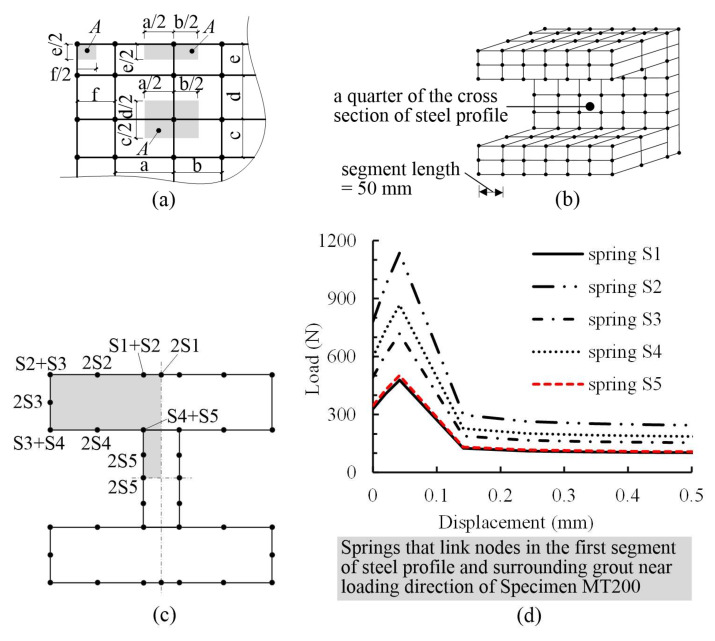
Implementation of bond stress-slip relationship for steel profile and grout in FEM models: (**a**) computation of *A*_i_; (**b**) segments of steel profile along the loading direction; (**c**) springs Sl~S5; and (**d**) force-displacement relationship of springs Sl~S5.

Two difficulties arose in the modeling. One was the simulation of the bond stress-slip relationship between the steel profile and grout. The other was the modeling of the shear force-slip relationship between the studs and surrounding grout. For the sleeves and grout, almost no slip was observed between them; therefore, a perfect bond was adopted. To resolve the first difficulty, it is believed that the bond action along the loading direction is dominant compared to that in the other two directions (i.e., circumferential and radial directions). Studies reveal that the bond behavior between the steel profile and concrete is affected by many factors including the thickness of concrete cover, transverse reinforcement ratio, anchorage length of steel profile, and concrete strength [28]. In particular, the bond stress-slip relationship depends on the location and position of the steel profile, which means the distance to the loading end of the steel profile, and flanges / web of steel profile, respectively. However, bond behavior between steel profile and grout is not available in the literature to the best of the authors’ knowledge. Thus, the bond stress–slip relationship between steel profile and concrete proposed by Yang [29] using push-out tests was adopted for that of steel profile and grout. As usual, the bond stress–slip relationship was implemented using spring elements in the software. To do this, two steps were adopted. In the first step, an imbedded steel profile was divided into certain number of segments along the loading direction. In this study the segment length was uniformly taken as 50 mm, as schematically presented in Figure 14b. For each segment, the force–deformation relationship for spring elements is expressed as Equation (2):

(2) $$F=\tau \left(s\right)\times A_{i}$$

where *F* denotes spring force, *τ* means bond stress which depends on the slip between steel profile and grout, *s*, and *A*_i_ represents subordinate area of node *i*, and is illustrated in Figure 14a. As a result, for a specific segment of steel profile, five types of springs (i.e., S1~S5) were generated on the surface of steel profile according to Equation (2) and indicated in Figure 14c in a quarter of the cross section of steel profile. As an example, Figure 14d depicts the force-deformation relationship of five springs linking the nodes in the first segment of steel profile and the surrounding grout near the opening end of Specimen MT200. Actually, the sharp of these curves were a little bit similar to the curve sharp of the well-known bond stress-slip relationship between deformed bar and concrete. These springs were modeled using a nonlinear spring model (*MAT_SPRING_GENERAL_NONLINEAR) provided by the software. In the second step, different segment resulted in different groups of five springs, which were used in the FEM models of the splices.

**Figure 15:**
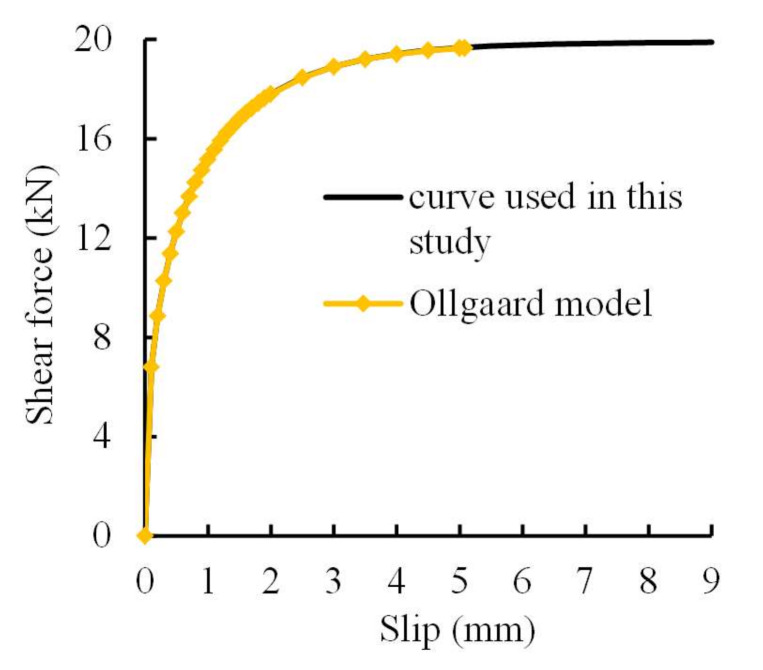
Shear force-slip curves for studs used in this study and proposed by Ollgaard [30].

**Figure 16:**
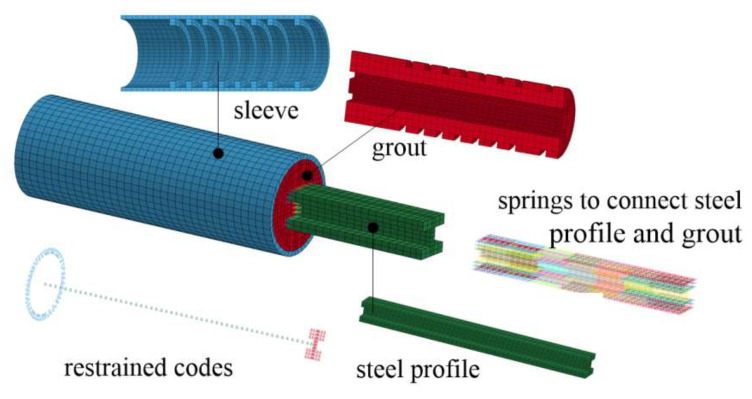
FEM model for Specimen RT350.

For the second difficulty, the often used shear force-slip relationship for studs and concrete proposed by Ollgaard [30] was adopted for that of the studs and grout, and is expressed in Equations (3) and (4):

(3) $$P/P_{u,\mathrm{st}}={\left(1-e^{-0.71s}\right)}^{0.4}$$

(4) $$P_{u,\mathrm{st}}=0.5A_{s}\sqrt{E_{c}f_{c}}\leq A_{s}f_{u}$$

where *P* and *s* denote shear force and slip, respectively; *P*_u,st_ refers to the ultimate shear resistance of studs; *A*_s_ and *f*_u_ are the cross sectional area of studs and the ultimate strength of the steel used for studs, respectively; *E*_c_ and *f*_c_ represent elasticity modulus and compressive strength of grout, respectively. Evidently, the curve of Equations (3) and (4) depend on the ultimate slip, which widely varies in the literature, e.g., 5.08 mm [30], 1.25 mm [31], and more than 10 mm [32]. In this study, the ultimate slip was taken as 9 mm. Figure 15 compares the shear force-slip curves for studs used in this study and that proposed in [30], resulting in minor difference. Consequently, nonlinear spring elements were used to describe the shear force–slip behavior in FEM models. Finally, Figure 16 illustrates the FEM model built for Specimen RT350 as an example, including 15,454 elements and 1,002,804 nodes.

### 4.2. Model Verification

**Figure 17:**
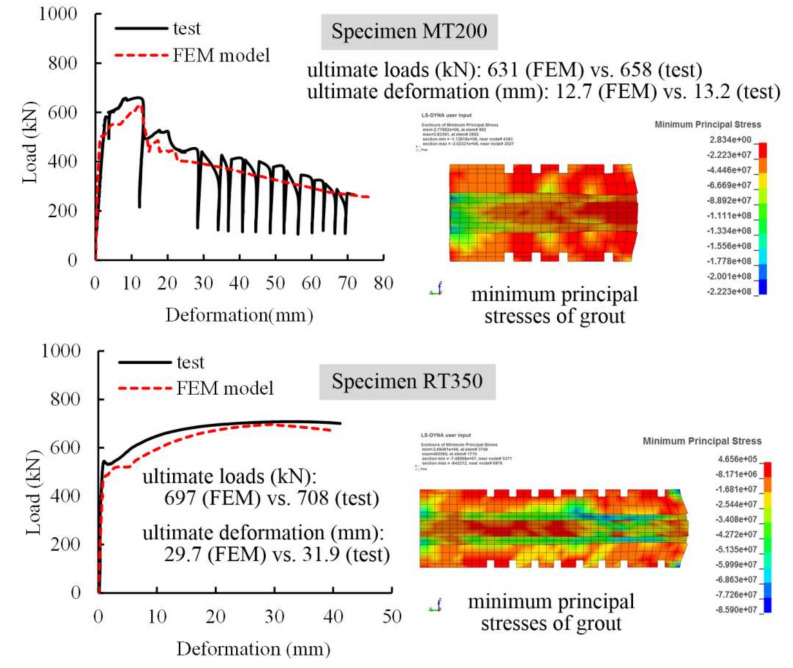
Load-deformation curves of Specimens MT200 and RT350 using FEM models and test data.

Numerical results were verified against test results using Specimens MT200 and RT350 because they represented two typical failure modes, i.e., fracture and pull-out of steel profile, respectively. In computation, both specimens were monotonically loaded, resulting in identical failure modes to those observed in tests. Figure 17 compares the load-deformation curves of Specimens MT200 and RT350 using FEM models and test data, and proper agreement was achieved for Specimens RT350. However, for Specimen MT200 the curve profile in test was properly depicted and a fluctuation of the curve in test was not simulated. This is because the FEM model could not well capture the repeated failure and compaction of local grout clung to studs that actually occurred in test. In addition, minimum principal stresses illustrated in Figure 17 were far more than the compressive strengths of grout under uniaxial loading, indicating appropriate simulation of the confining effect of grout caused by the sleeves. Finally, close results using FEM models and in tests were obtained in terms of strain development of the steel profiles, strain development of the sleeves, and deformation contributions for the specimens at ultimate loads. These results are not presented for brevity. Thus, the FEM models are reliable and can be used to perform parametric studies.

### 4.3. Parametric Studies

Splice behavior was influenced by a quantity of parameters including sleeve thickness, grout strength, offset of steel profile, and misalignment of steel profile. Table 7 presents the designed specimens for parametric studies using FEM models. In each case of parametric studies, a reference specimen was selected. Only the value of one parameter of the reference specimen varied, while the others remained unchanged. All specimens were monotonically loaded to failure.

#### 4.3.1. Sleeve Thickness

**Figure 18:**
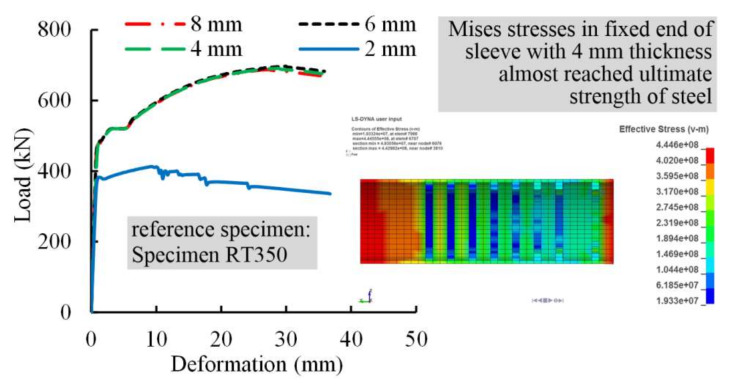
Load-deformation curves for specimens with different sleeve thicknesses using FEM models.

Previous studies revealed that increasing sleeve thickness leads to enhance the confining effect in the radial direction, and further increases the bond action between steel profile and grout [33]. In the tests, the sleeve thickness was conservatively taken as 8 mm for all specimens. In parametric study, Specimens RT350 was taken as reference specimen and sleeve thickness was individually set to 2, 4, 6, and 8 mm. Figure 18 illustrates load-deformation curves for these specimens and critical results are presented in Table 7. Results found that, (1) the failure mode of the specimen with sleeve thickness of 2 mm was sleeve fracture; (2) the failure mode of specimens with sleeve thicknesses of 4, 6, or 8 mm was steel profile fracture, and their ultimate loads almost remained unchanged; and (3) the stress in the fixed end of the sleeve with 4 mm thickness almost reached the ultimate strength of steel, and the splice was at the risk of failure. However, splices with sleeves of 6 and 8 mm thicknesses behaved elastically. As a result, the proper sleeve thickness for the splice in this case was recommended to range from 4 to 6 mm.

#### 4.3.2. Grout Strength

**Figure 19:**
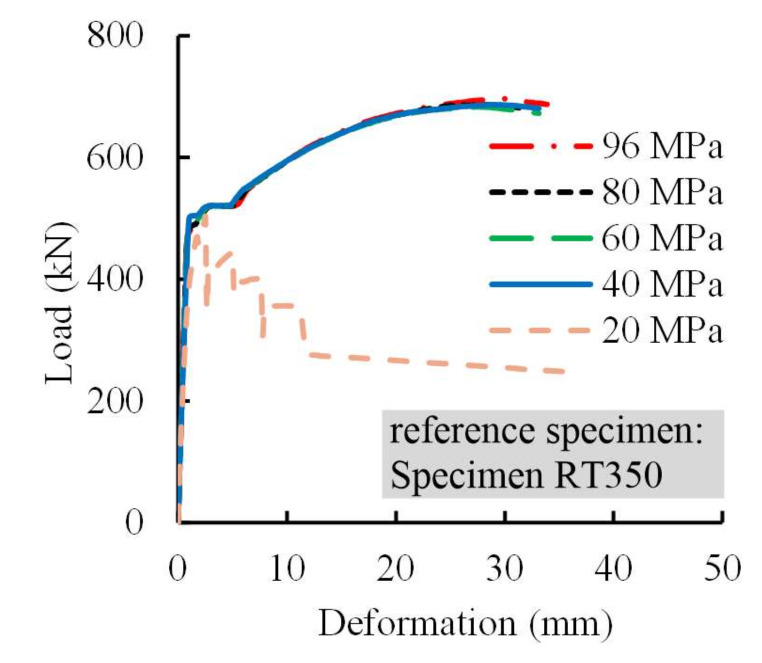
Load-deformation curves for specimens with different grout strengths using FEM models.

Grout strength affects the failure mode of splices, which can be demonstrated by investigating the responses of splices with different grout strengths under loading. For reference specimen RT350, the compressive strengths of the grout were individually taken as 96, 80, 60, 40, and 20 MPa. The currently used grout in construction in China commonly ranged from 80 to 95 MPa [23]. Figure 19 illustrates the load-deformation curves for these specimens, and the critical results are presented in Table 7. It was revealed that the splice failed due to steel profile pull-out when the compressive strength of 20 MPa was used for the grout, resulting in a fairly low ultimate resistance. However, the failure mode of steel profile fracture occurred when the splices’ grout had a compressive strength of more than 40 MPa. In these cases, their ultimate loads and ultimate deformations were close to each other. This implied that increasing compressive strengths of the grout had little effect on the splice performance if the compressive strength threshold was exceeded.

#### 4.3.3. Offset

**Figure 20:**
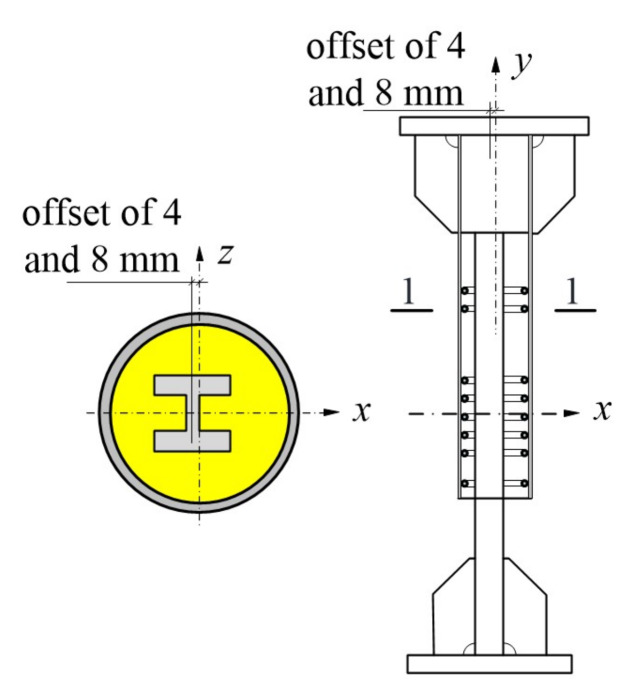
Offset of profile steel from the *z* axis.

Offset and misalignment occasionally occurred during splice manufacturing and at the construction site. For reference specimen RT350, Figure 20 illustrates the steel profile moving along the *x* axis (strong axis) and offsetting from the *z* axis (weak axis) by 4 and 8 mm, i.e., 0.324 and 0.648 times the radius of gyration with respect to the *z* axis. Critical results are presented in Table 7. Results indicated that the ultimate loads and ultimate deformation were almost unaffected by offset in these cases. Similar results were also observed in [12] using grouted sleeves for splicing rebars.

#### 4.3.4. Misalignment

**Figure 21:**
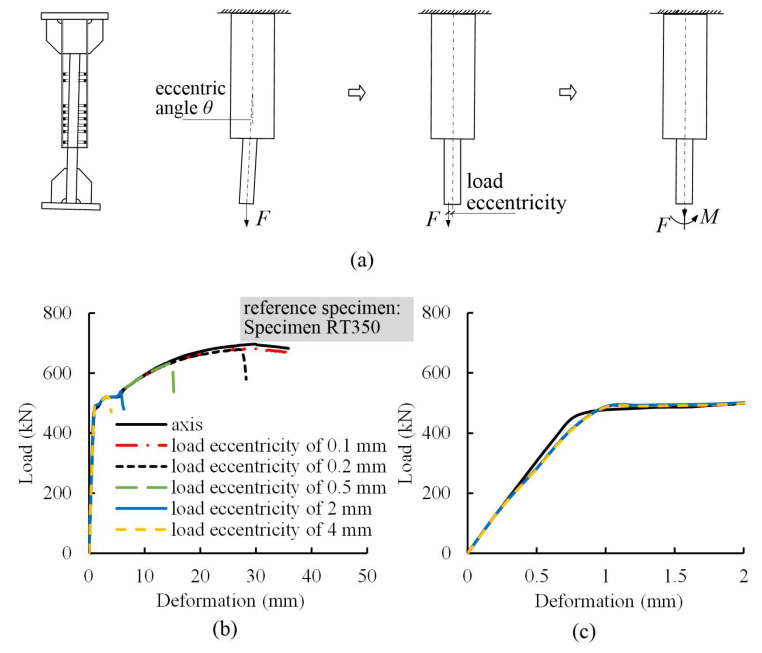
(**a**) Misalignment of steel profile; (**b**) load-deformation curves for specimen with different load eccentricities using FEM models; and (**c**) elastic parts of these curves.

Figure 21 illustrates the misalignment of steel profile in reference Specimen RT350. The eccentric angles were set to 0.00014, 0.00029, 0.00072, 0.00288, and 0.00576 rad, resulting in load eccentricities at the steel profile end of 0.1, 0.2, 0.5, 2, and 4 mm, which were 0.008, 0.016, 0.040, 0.162, and 0.324 times the radius of gyration with respect to the *z* axis, respectively. The eccentricity of loading was approximately equivalent to the combined action of a tensile force and a moment imposed at the steel profile end. Figure 21 illustrates load–deformation curves for these specimens and critical results are presented in Table 7. It was revealed that the misalignment had a pronounced effect on splice behavior. The ultimate load and ultimate deformation decreased as the misalignment increased, although the elastic behavior of these specimens kept almost unchanged. This was attributed to a non-uniform stress distribution along the cross section of the steel profile, which led to the premature fracture of splices [10].

## 5. Conclusions

Seven grouted sleeve splices for the steel profile were designed and tested under various loading schemes of MT, RT, HS, and LS to investigate their tensile behavior. Parametric studies using FEM models were performed to understand the influence of sleeve thickness, grout strength, offset of steel profile, and misalignment of steel profile on splice behavior. Based on the experimental and numerical results, the following conclusions can be drawn:

(1) Splices with an appropriate anchorage length of the steel profile behaved well under loading schemes of RT, HS, and LS. The skeletons of the load-deformation curves, ultimate strengths and relative elongations at ultimate loads were similar to each other. Minor residential deformation and almost no pinching effect were observed.
(2) Proper sleeve thickness could be determined using the numerical approach to optimize the configuration of splices.
(3) Splice performance was insignificantly affected by the offset of the steel profile and the compressive strengths of grout that exceeded the threshold value. However, splice behavior was significantly affected by the misalignment of the steel profile.

Based on the limited test data and numerical results in this study, grouted sleeve splices for the steel profile presented appropriate performance with potential application in the proposed precast reinforced concrete shear wall structures. However, further research is needed to investigate the tensile behavior of the splices with a variety of parameters comparable to those in practice. In addition, subsequent studies on the structures are ongoing by the authors to identify their direct shear and seismic performance.

## Figures and Tables

**Table 1:** Specimen details.

Group No.	Specimen No.	Cross Section of Steel Profile (mm)	Cross Section of Sleeve (mm)	Embedded Length of Steel Profile, *L* (mm)	Number of Steel Ring Ribs	Number of Steel Studs	Loading Scheme
1	MT500	I46 × 46 × 12 × 8	Φ121 × 6	500	10	8	MT
	MT400			400	10	6	MT
	MT300			300	8	4	MT
	MT200			200	4	2	MT
2	RT350	I46 × 46 × 12 × 8	Φ121 × 6	350	8	6	RT
	HS350			350	8	6	HS
	LS350			350	8	6	LS

**Table 2:** Material properties for steel profile and sleeve steel [21].

Material	Group No.	Yield Strength (MPa)	Ultimate Strength (MPa)	Elasticity Modulus (GPa)	Yield Strain (%)	Ultimate Strain (%)
Steel profile	1	437.4	580.0	215	0.203	16.6
	2	410.0	543.7	222	0.185	14.5
Steel for sleeves	1	420.7	624.0	227	0.185	14.5
	2	445.2	599.7	229	0.194	13.2

**Table 3:** Material properties for grout [22].

Group No.	Compressive Strength (MPa)	Flexural Strength (MPa)	Elasticity Modulus (×10^4^ MPa)	Tensile Strength (MPa)
1	68.0	12.7	-	-
2	95.8	17.4	3.89	7.44

Note: Elasticity modulus and tensile strength of the grout in group 1 were not measured.

**Table 4:** Loading procedures for different loading schemes.

Loading Scheme	Loading Procedure
Monotonic tensile loading (applied for Specimens MT500, MT400, MT300 and MT200)	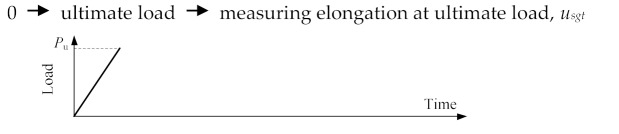
Repeated tensile loading (applied for Specimen RT350)	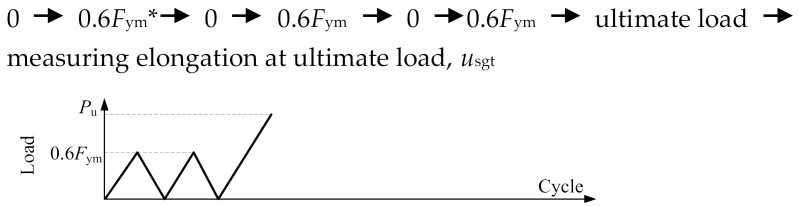
Cyclic loading at high stress (applied for Specimen HS350)	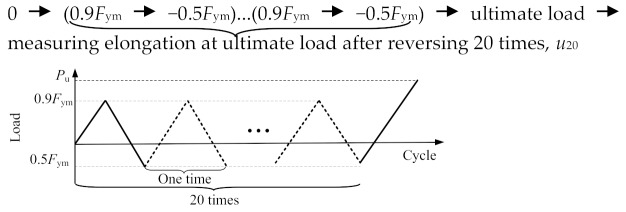
Cyclic loading at large strain (applied for Specimen LS350)	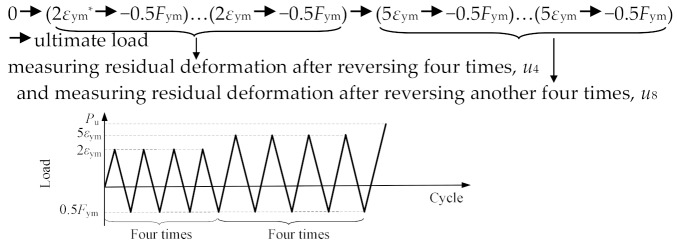

* Note: *F*_ym_ and *ε*_y__m_ denote force corresponding to yield strength and yield strain of steel profile, respectively.

**Table 5:** Primary results for specimens in the first test group.

Specimen No.	Measured Cross Sectional Area of Steel Profile (mm^2^)	Yield Loads (kN)	Yield Strength (MPa)	Ultimate Loads (kN)	Ultimate Strength (Mpa)	Relative Elongations at Ultimate Loads, *δ*_sgt_ (%)	Failure Mode of Steel Profile
MT500	1328	610	460	783	589	3.8	Fracture
MT400	1328	616	464	791	596	3.4	Fracture
MT300	1304	600	460	772	592	5.2	Fracture
MT200	1280	602	470	658	514	2.2	Pull-out

**Table 6:** Primary results for specimens in the second test group.

Specimen No.	Measured Cross Sectional Area of Steel Profile (mm^2^)	Yield Loads(kN)	Yield Strength (Mpa)	Ultimate Loads (kN)	Ultimate Strength (Mpa)	Relative Elongations at Ultimate Loads, *δ*_sgt_ (%)	Failure Mode of Steel Profile
RT350	1237	531	430	708	572	6.1	Fracture
HS350	1311	540	412	721	550	6.2	Fracture
LD350	1240	546	440	718	580	6.6	Fracture

**Table 7:** Designed specimens for parametric studies using FEM models.

Parameter	Reference Specimen	Value for Parameter	Failure Mode	Yield Load (kN)	Ultimate Load (kN)	Ultimate Defor-mation (mm)	*δ*_sgt_ (%)
Sleeve thickness	Specimen RT350	2 mm	sleeve fracture	-	413	9.2	1.7
		4 mm	steel profile fracture	520	697	28.7	5.4
		6 mm	steel profile fracture	520	697	29.7	5.6
		8 mm	steel profile fracture	520	697	29.7	5.6
Grout strength	Specimen RT350	20 MPa	steel profile pull-out	-	503	2.5	0.5
		40 MPa	steel profile fracture	520	687	28.9	5.4
		60 MPa	steel profile fracture	520	683	26.9	5.0
		80 MPa	steel profile fracture	520	787	26.0	4.9
		96 MPa	steel profile fracture	520	697	29.7	5.6
Offset	Specimen RT350	0 mm	steel profile fracture	520	697	29.7	5.6
		4 mm	steel profile fracture	520	697	30.5	5.7
		8 mm	steel profile fracture	520	697	30.3	5.6
Mis-alignment	Specimen RT350	0 rad	steel profile fracture	520	697	29.7	5.6
		0.008 rad	steel profile fracture	520	683	29.0	5.4
		0.016 rad	steel profile fracture	520	678	27.0	5.1
		0.040 rad	steel profile fracture	520	633	14.7	2.8
		0.162 rad	steel profile fracture	520	539	5.7	1.1
		0.324 rad	steel profile fracture	520	521	3.5	0.7

## Nomenclature

*A*_i_	Subordinate area of node *i* of steel profile
*A*_s_	Cross sectional area of studs
*E*_c_	Elasticity modulus of grout
*F*	Spring force
*f*_c_	Compressive strength of grout
*f*_u_	Ultimate strength of the steel used for studs
*F*_ym_	Force corresponding to yield strength of steel profile
*h*	Cross sectional height of steel profile
HS	Cyclic loading at high stress
MT	Monotonic tensile loading
*L*	Embedded length of steel profile
*L*_g_	Gauge length of specimens
LS	Cyclic loading at large strain
*P*	Shear force of studs
*P*_u_	Ultimate shear resistance of specimens
*P*_u,st_	Ultimate shear resistance of studs
RT	Repeated tensile loading
*s*	Slip between steel profile and grout
*u*_4_	Residual deformation after reversing four times of a LS-series splice
*u*_8_	Residual deformation after reversing eight times of a LS-series splice
*u*_20_	Elongation at ultimate load after reversing 20 times of a HS-series splice
*u*_sgt_	Elongations at ultimate loads within gauge length
*δ*_sgt_	Relative elongation at ultimate loads within gauge length
ε_y__m_	Yield strain of steel profile
*τ*	Bond stress due to slip between steel profile and grout
